# Altered Fatty Acid Oxidation in Lymphocyte Populations of Myalgic Encephalomyelitis/Chronic Fatigue Syndrome

**DOI:** 10.3390/ijms24032010

**Published:** 2023-01-19

**Authors:** Jessica Maya, Sabrina M. Leddy, C. Gunnar Gottschalk, Daniel L. Peterson, Maureen R. Hanson

**Affiliations:** 1 Department of Molecular Biology and Genetics, Cornell University, Ithaca, NY 14850, USA; 2 Simmaron Research, Incline Village, NV 89451, USA; 3 Sierra Internal Medicine, Incline Village, NV 89451, USA

**Keywords:** myalgic encephalomyelitis, chronic fatigue syndrome, beta-oxidation, fatty acid oxidation, T cells, Natural Killer cells, immunometabolism

## Abstract

Myalgic Encephalomyelitis/Chronic Fatigue Syndrome (ME/CFS) is a disabling multisystem illness in which individuals are plagued with fatigue, inflammatory symptoms, cognitive dysfunction, and the hallmark symptom, post-exertional malaise. While the cause of this disease remains unknown, there is evidence of a potential infectious component that, along with patient symptoms and common onsets of the disease, implicates immune system dysfunction. To further our understanding of the state of ME/CFS lymphocytes, we characterized the role of fatty acids in isolated Natural Killer cells, CD4+ T cells, and CD8+ T cells in circulation and after overnight stimulation, through implicit perturbations to fatty acid oxidation. We examined samples obtained from at least 8 and as many as 20 subjects for immune cell fatty acid characterization in a variety of experiments and found that all three isolated cell types increased their utilization of lipids and levels of pertinent proteins involved in this metabolic pathway in ME/CFS samples, particularly during higher energy demands and activation. In T cells, we characterized the cell populations contributing to these metabolic shifts, which included CD4+ memory cells, CD4+ effector cells, CD8+ naïve cells, and CD8+ memory cells. We also discovered that patients with ME/CFS and healthy control samples had significant correlations between measurements of CD4+ T cell fatty acid metabolism and demographic data. These findings provide support for metabolic dysfunction in ME/CFS immune cells. We further hypothesize about the consequences that these altered fuel dependencies may have on T and NK cell effector function, which may shed light on the illness’s mechanism of action.

## 1. Introduction

Myalgic encephalomyelitis/chronic fatigue syndrome (ME/CFS) is a debilitating disease estimated to isolate approximately 65 million people worldwide to a life of pain, severe fatigue, cognitive impairments, gastrointestinal symptoms, post-exertional malaise, and orthostatic intolerance [1,2,3]. Further, people with ME/CFS frequently exhibit immune-related symptoms that resemble influenza, such as headaches, muscular pain, tender lymph nodes, and fever. The onset of the disease can be gradual or abrupt, and the severity of the disease can vary, with some individuals completely house- or bed-bound for decades without relief [3]. In the past, this underfunded and misunderstood disease has occurred in clustered outbreaks, but most cases in recent years have been sporadic [4]. This knowledge and other substantial evidence imply an infectious agent at work, but its identity remains controversial [5,6,7,8]. Currently, there is no FDA-approved drug to treat individuals with ME/CFS [9].

The infectious nature of ME/CFS has prompted investigations into the immune system, related cells, and functional capabilities. Numerous studies of cytokine production and immune cell population frequencies in ME/CFS have been conducted but frequently conflict due to the variability of immune characteristics based on age, sex, health status, genetics, microbiomes, nutrition, and other undetermined factors [10]. Nevertheless, correlations have been reported between the illness duration or disease severity and cytokines such as TNF-α, TGF-β, IL-1α, IL-1β, IL-4, IL-6, and IFN-γ in ME/CFS plasma [11,12,13,14,15,16]. Immune cell population findings have shown decreased regulatory T cell (Treg) levels, increased effector memory CD8+ T cells, and decreased terminally differentiated effector CD8+ T cells [17,18]. Functionally, ME/CFS Natural Killer (NK) cell and CD8+ T cell cytotoxicity is diminished compared to healthy subjects [19,20,21,22,23,24]. Although these studies demonstrate immunological dysfunction in ME/CFS lymphocytes, there is still much to learn about the causes and consequences of these findings.

Meanwhile, ME/CFS metabolic markers have drawn interest in general and as prospective biomarkers for diagnosis, particularly due to symptoms such as post-exertional malaise and fatigue. Prior reports in ME/CFS have found evidence of oxidative stress, lower ATP levels, and decreased coenzyme Q10, necessary for ATP production in the electron transport chain [25,26,27]. Additionally, an array of metabolomic studies from ME/CFS plasma, serum, and urine samples have revealed metabolite alterations related to the metabolism of amino acids, nucleotides, glucose, nitrogen, hormones, glutamate, and fatty acids [26,28,29,30,31,32,33,34,35]. Metabolite fluctuations in the extracellular environment have likely influenced intracellular metabolism, a tightly regulated network that, when disturbed, can cause cellular impairment.

These findings prompted us to explore ME/CFS immune cell metabolism, a complex and carefully controlled system in healthy individuals. Metabolic remodeling occurs during a cell’s lifespan, altering itself to promote specific functions and comply with energetic demands. At first, circulating naïve T cells and NK cells will produce energy through oxidative phosphorylation (OXPHOS), favoring fatty acid oxidation (FAO) to meet the low metabolic needs of these lymphocyte populations [36,37,38,39]. Once activated, cells require a quick and robust immune response. By upregulating OXPHOS and glycolysis, immune cells can produce large amounts of energy while simultaneously synthesizing macromolecules and intermediates from glycolysis that are essential for effector molecule and biomass production that will directly or indirectly kill infected cells [37,38]. Following the immune response, some effector cells will differentiate into memory cells and increase mitochondrial mass, expand spare respiratory capacity, and downregulate glycolysis to rely on FAO for the essential long-lived maintenance and potential reactivation of this subset [37,40]. In addition to these populations, other subsets of T cells have been found to rely on distinctive metabolic pathways, typically dependent on their suppressive or effector functional state [41,42]. These studies highlight the importance of investigating metabolic pathways in isolated lymphocyte populations to determine specific energy abnormalities and the unique functional consequences of such alterations.

Prior research into ME/CFS immunometabolism has investigated more general cell populations, such as total peripheral blood mononuclear cells (PBMCs), which include dendritic cells, monocytes, T cells, B cells, and NK cells. For instance, ME/CFS PBMC bioenergetic assays have revealed reduced mitochondrial respiration, reduced glycolysis, no difference in mitochondrial complex activity, lower mitochondrial coupling efficiency, and altered pyruvate dehydrogenase and CoA metabolism, suggesting inadequate ATP production and FAO dysregulation [43,44,45,46]. Analysis of the proteomes of PBMCs indicates elevated levels of enzymes involved in ketone body metabolism and a greater expression of acyl-CoA dehydrogenases in ME/CFS subjects [47]. Further, another research team reported upregulated levels of enzymes that mediate fatty acid oxidation, such as very long chain specific fatty acid enzyme components and, similar to the proteomic assay, acyl-CoA dehydrogenases, in ME/CFS immortalized cultured lymphoblasts [48,49].

Isolated immune cell population metabolic studies have been limited. A 2019 pilot study on NK cell metabolism reported a decreased glycolytic reserve in ME/CFS subjects [50]. Our group has previously focused on glycolysis and oxidative phosphorylation studies in T cells, wherein we discovered lower rates of glycolysis and mitochondrial membrane potential, along with dysregulated cytokine associations and no changes in mitochondrial respiration in ME/CFS T cells [51]. The metabolic profile of CD4+ and CD8+ T cells that we observed is consistent with a chronic viral infection and is commonly seen in T cell exhaustion. According to past studies on long-term antigen exposure that result in this exhausted state, the metabolic requirements for T cell activity are permanently changed to favor this suppression of immune response [52]. Particularly, glycolysis is inhibited, and FAO is promoted in T cells in chronic antigen-stimulating environments [53].

We still do not know the full depth of metabolic remodeling that occurs in specific ME/CFS lymphocytes. There have been no studies that have probed FAO dependencies in isolated ME/CFS immune cell subsets. We know that FAO usage is particular to certain cell states, and deviations from appropriate metabolic pathways in immune cells can dictate cell function, alter cell differentiation, facilitate disease initiation, and influence illness management. For example, an investigation of IFN-γ production in glucose-limiting media revealed that effector T cell functionality could be determined by the ratio of glycolysis to fatty acid metabolism [54]. In NK cells, increased environmental FA levels and subsequent FAO escalation impaired NK cell function, decreasing cytokine production in a human lymphoma study [55]. Studying alternative fuel utilization in ME/CFS immune cells can provide insight into the onset and progression of the disease and inform researchers of prospective treatment targets.

To determine the role of fatty acid metabolism and underlying cellular components in isolated ME/CFS immune cell populations, we have carried out metabolic assays in ME/CFS and healthy control CD56+ NK cells, CD4+ T cells, and CD8+ T cells at rest and after stimulation. We performed extracellular flux analysis accompanied by a fatty acid oxidation drug panel to determine fatty acid oxidation utilization under high energy demands in isolated lymphocytes. In addition, we have used flow cytometry to analyze a fluorescently tagged fatty acid analog and two pertinent fatty acid transporters to quantify specific energy dynamics in these cell populations. By including additional cell surface markers in this assay, we further characterized FAO in more specific T cell subsets, such as naïve, effector, and memory cells. Lastly, we obtained survey information from the study participants and analyzed our data for correlations. We hypothesize that fatty acid oxidation is increased in specific immune cell types, contributing to improper immune cell function in ME/CFS.

## 2. Results

### 2.1. Study Population Characteristics and Sample Collection

Most subjects in this study were recruited by Dr. Daniel Peterson in Incline Village, NV. Other subjects included in this study were identified by physicians through the NIH-funded Cornell Collaborative ME/CFS Center at Ithaca College, Weill Cornell Medicine, and EVMED (Supplementary Table S1) or underwent cardiopulmonary exercise testing by Dr. Betsy Keller at Ithaca College (blood was collected prior to exercise testing). Each cell population (NK cells, CD4+ T cells, and CD8+ T cells) was comprised of a range of 28–44 ME/CFS subjects and 26–39 healthy controls, while each experiment within these cell populations involved 8–20 individuals per condition. The exact subject population for each result can be found in the respective figure caption. For each cell type, the age and sample size distribution of the two cohorts were comparable (Table 1). Each cell type included both males and females, and the race of most participants was White, while the ethnicity of all subjects was either non-Hispanic or unknown (Supplementary Table S1). Individuals with ME/CFS reported an average illness duration of 16 to 20 years within each cell population cohort, ranging from 1 to 54 years for all patients in this study (Table 1). Of the patients who reported their type of illness onset, 7–12 reported a gradual development of ME/CFS, while 11–13 described a sudden onset of the disease; the remaining participant’s onset type was unknown (Table 1). Although many participants were asked to submit a list of their current prescription drugs and dietary supplements, it was not feasible to control for medications.

Many of the subjects completed a Bell Activity Scale that ranges from 0 to 100, where a score of 0 indicates severe disability or impairment while 100 represents a healthy individual [56]. In the three cohorts, patients reported an average score of 30.6–37, whereas healthy controls scored a mean of 96.5–98.7 on this questionnaire (*p* < 0.001) (Table 1 and Supplementary Table S1). Additionally, most participants completed the 36-item short-form survey (SF-36), which can be used to calculate a variety of physical health, mental health, and quality-of-life measurements. In this assessment, 100 indicates good health, while 0 reflects severe disability [57]. ME/CFS patients had lower scores on all dimensions of this survey, particularly within the physical function variables (Table 1 and Supplementary Table S1). While most of the measurements were statistically significant between patient and control groups, it should be noted that within both T cell cohorts, the emotional role and mental health components were not statistically significantly different when tested with a Wilcoxon rank-sum test (Supplementary Table S1, highlighted). These dimensions measure role limitations due to mental health difficulties. Notwithstanding, when compared to healthy controls in this study, the overall ME/CFS patient survey metrics showed a significant impairment, demonstrating that this cohort clearly displays common indicators of the disease.

**Figure 1:**
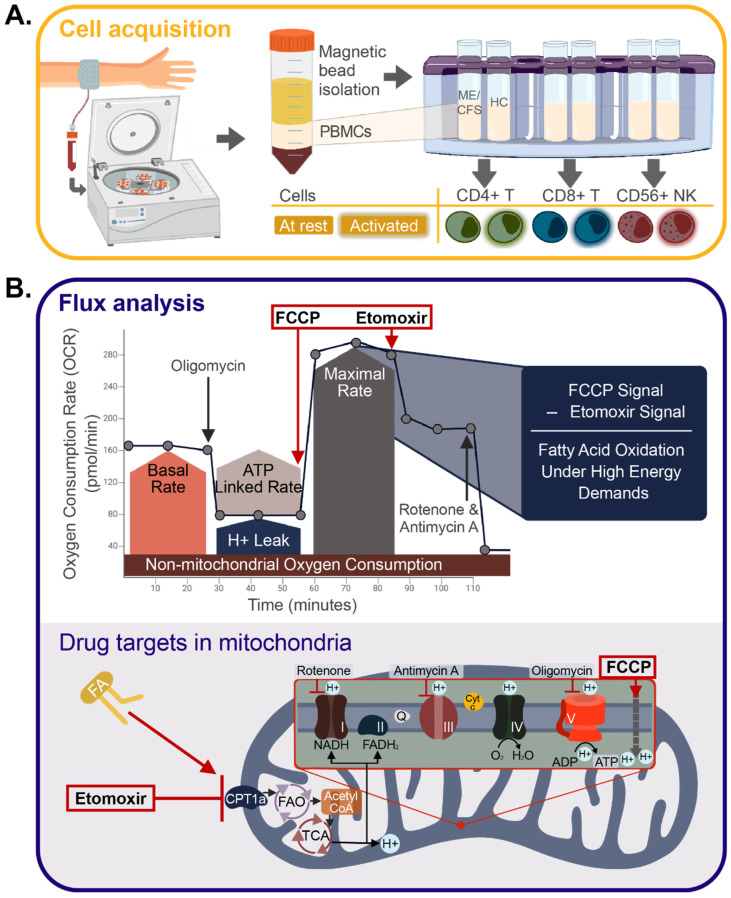
Immune cell isolation study design and extracellular flux analysis. (**A**) Cell acquisition approach, where peripheral blood mononuclear cells (PBMCs) were isolated from whole blood taken from both healthy controls (HC) and individuals with ME/CFS. Magnetic bead isolation was performed using positive selection, starting with CD56+ Natural Killer (NK) cells, followed by CD8+ T cells, and, finally, CD4+ T cells. Isolated immune cells were activated 24 h before the experiment with ⍺CD3/⍺CD28 and IL2 for T cells and IL15/IL12 for NK cells. (**B**) Seahorse drug injection strategy, where the *y*-axis represents the rate of oxygen consumption in pmol/minutes, and the *x*-axis is the time during the assay in minutes. A modified Mito Stress test drug injection strategy was performed on isolated immune cell types. Etomoxir, an inhibitor of fatty acid oxidation, was included in post-FCCP measurements to calculate the fatty acid contribution of each cell type under high energy demands. Included in panel B is a visual representation of each drug target within the mitochondria (FA = fatty acid, FAO = fatty acid oxidation, TCA = tricarboxylic acid cycle).

Peripheral blood mononuclear cells (PBMCs) were isolated shortly after blood sample collection and subsequently shipped overnight on dry ice to Cornell University for storage in liquid nitrogen. Lymphocytes thawed from frozen stocks were separated into CD56+ NK cells, CD8+ T cells, and CD4+ T cells for all samples using positive selection magnetic bead kits (Figure 1A).

### 2.2. ME/CFS Natural Killer Cells Exhibit Higher Fatty Acid Utilization Compared to Healthy Cells

To investigate the contribution of fatty acids in patient and healthy control NK cells, we used an Agilent Seahorse Xfp extracellular flux analyzer with a modified Mito Stress Test (Figure 1B). This assay examines the metabolic rates of live cells over time while methodically injecting metabolic stimulators or inhibitors to identify the cells’ dependence on fatty acid oxidation. Figure 1B illustrates the results of this drug injection assay—the oxygen consumption rate (OCR) on the *y*-axis describes the mitochondrial component of metabolism, while the *x*-axis denotes the time points, with different drugs injected sequentially during the assay. Particularly, carbonyl cyanide p-triflouro-methoxyphenyl hydrazone (FCCP), an uncoupling agent that mimics a physiological energy demand by stimulating the respiratory chain to operate at maximum capacity, and etomoxir (Eto), a long-chain fatty acid oxidation inhibitor that blocks the mitochondrial membrane FA transporter, carnitine palmitoyl transferase 1 (CPT1a), are of consequence to the fatty acid measurements. The difference between these two rates (change in OCR) reflects the role of fatty acid oxidation in these cells during periods of increased energy demand, such as those present in sites of inflammation. This injection strategy was adapted from previous studies looking at effector and memory T cell fatty acid contribution, but the concentration of etomoxir was reduced to avoid off-target effects of this inhibiting agent [58,59,60,61].

For CD56+ NK cells, we assayed the change in OCR after overnight stimulations with IL12 and IL15, which increase NK cell proliferation and cytotoxicity, stimulate IFN-γ production, and induce a robust glycolytic metabolic response [62,63,64]. We occasionally lacked enough NK cells to include a subject in every flux analysis. This problem was due, in part, to the varied viability status of the PBMCs we received. Additionally, some subjects had a greater frequency of certain cell types than others, which is an expected limitation of working with human immune cells [10]. For these reasons, preference was given to stimulating samples of NK cells that had at least ~100,000 cells/sample to perform this assay.

**Figure 2:**
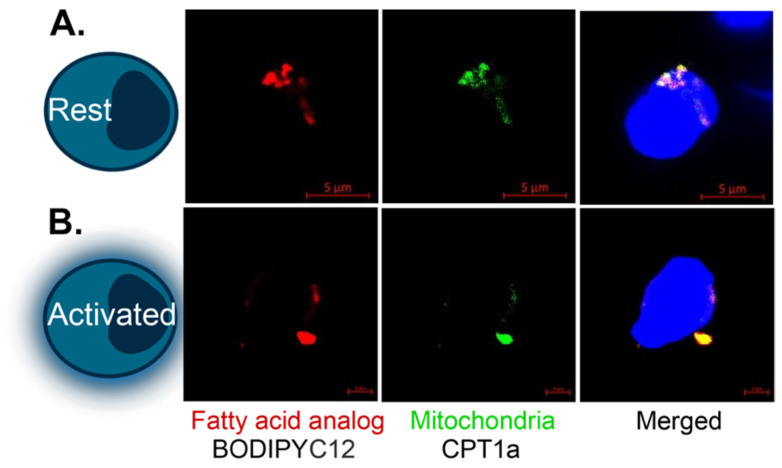
Colocalization of BODIPY and the mitochondria in T cells. Fatty acid analog (Bodipy 558/568 C12), CPT1a (Alexa Fluor 488), and Hoechst staining of representative (**A**) resting and (**B**) activated healthy control CD8+ T cells. This experiment was conducted 3–4 times for each condition and representative images are shown. Scale bars: (**A**) 5 μM and (**B**) 2 μM.

**Figure 3:**
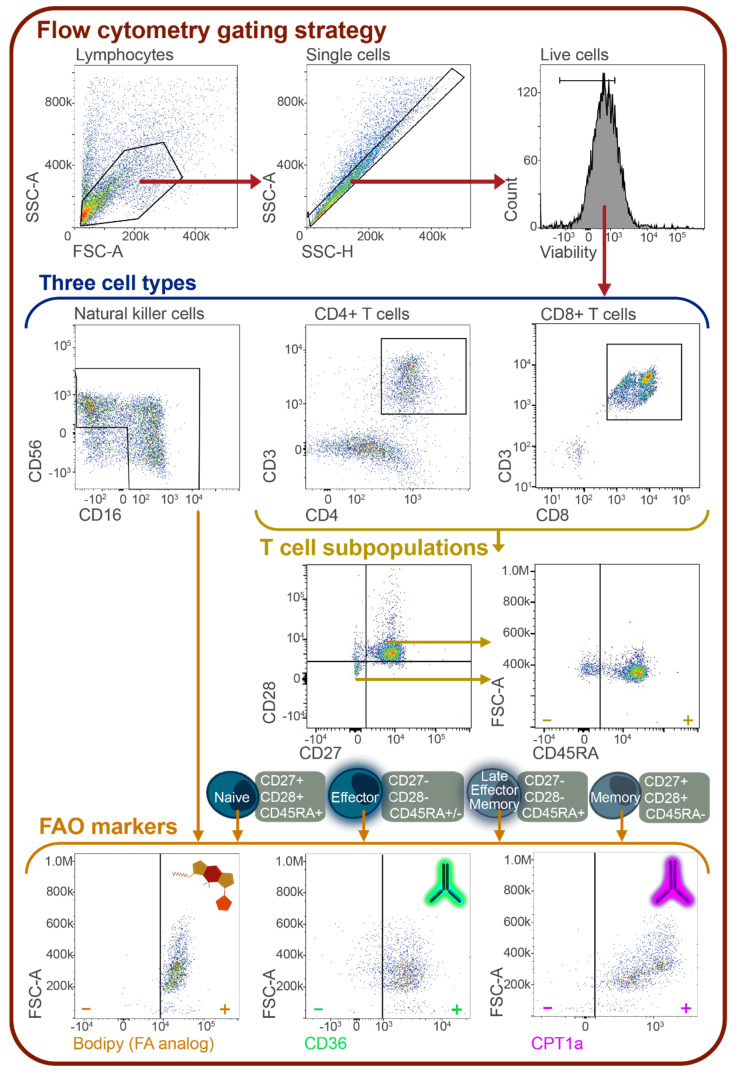
Flow cytometric cell population gating strategy for T and NK cells. All cells were gated by size, granularity, and viability using FSC-A, SSC-A, SSC-H, and eBiosciences Fixable Viability Dye eFluor 506. Isolated NK cells were gated to include CD56+CD16+, CD56+CD16-, and CD56-CD16+ cells. CD4+ T cells were gated for naïve (CD27+CD28+CD45RA+), effector (CD27-CD28-CD45RA+/-), late effector memory (CD27-CD28-CD45RA+), and memory (CD27+CD28+CD45RA-) cell populations, based on prior studies using these markers for cell population identification [65,66,67]. The same populations were identified in CD8+ T cells. Within each of these populations and in total CD8+ and CD4+ T cells, the mean and median fluorescence intensity (MFI) of BODIPY 558/568 C12, CPT1a, and CD36 were calculated. Specific antibodies and fluorophores used can be found in Supplementary Table S2.

The complete oxidation of fatty acids depends on several cellular components to function. Therefore, we delved further into this pathway’s mechanics to determine the abundance of CPT1a, the transporter enzyme mentioned above that controls the rate-limiting step of FAO, and CD36, a long-chain free fatty acid transporter located on the cell surface, via flow cytometry. Additionally, we incubated cells with a fluorescently tagged fatty acid analog (C12 Bodipy 568/568) and evaluated the abundance of this molecule within ME/CFS and healthy control NK cells using flow cytometry. This strategy allowed us to ascertain the absorption of an exogenous fatty acid, whereas flux analysis could only provide information on non-specific long-chain fatty acid uptake. We confirmed that this supplied fatty acid was reaching the mitochondria by microscopy (Figure 2). After gating single, live NK cells, we calculated the mean or median fluorescence intensity for each of these three fatty acid oxidation markers in circulating and stimulated NK cells (Figure 3). 

**Figure 4:**
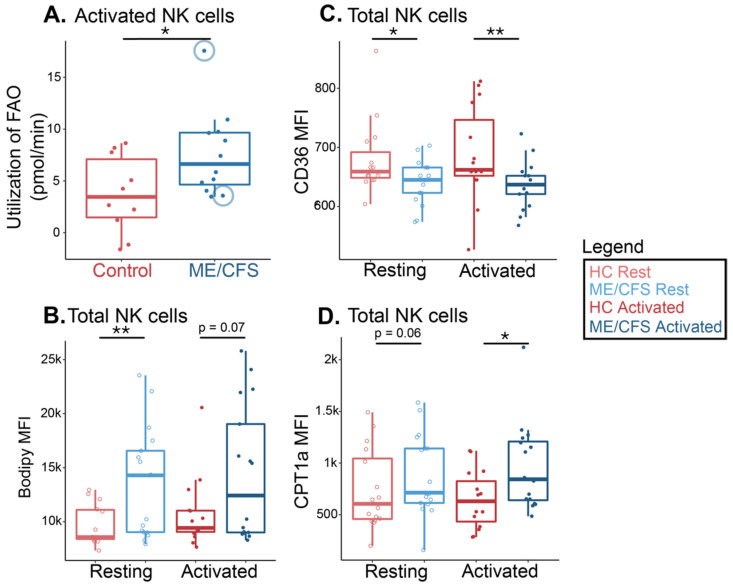
ME/CFS NK cells increased fatty acid oxidation compared to healthy controls. (**A**) Fatty acid oxidation measurements from a Seahorse extracellular flux analysis of stimulated NK cells (*n* = 10 healthy control samples; *n* = 12 ME/CFS samples). Each dot represents the change in oxygen consumption rate (pmol/min) under high energy demands following the injection of a fatty acid oxidation inhibitor (etomoxir) in one sample, with an average of 2–3 technical replicates. The blue circles around two of the data points indicate one patient at two different time points (**B**) Mean fluorescence intensity of a supplied fatty acid (Bodipy) in resting and activated NK cells (*n* = 14 healthy control samples at rest; *n* = 17 ME/CFS samples at rest; *n* = 15 healthy control samples after activation; *n* = 17 ME/CFS samples after activation, [*n* = 14/17/15/17]). (**C**) Median fluorescence intensity of a fatty acid cell membrane transporter, CD36, in resting and activated NK cells (*n* = 15/17/15/17). (**D**) Median fluorescence intensity of a fatty acid mitochondrial transporter, CPT1a, in resting and activated NK cells (*n* = 16/17/15/16). Box plots represent the median ± 25th and 75th quartiles. Whiskers represent 1.5× the interquartile ranges. Outliers are values outside the whisker range. Healthy control and ME/CFS data points are light (resting) or dark (activated) red or blue, respectively. For Seahorse data, * *p* < 0.05 by Wilcoxon rank-sum test. For flow cytometry data, * *p* < 0.05, ** *p* < 0.01 by Kruskal–Wallis followed by Dunn’s test with FDR-based multiple testing correction.

Natural Killer cells showed a significant increase in fatty acid oxidation utilization under high energy demands in ME/CFS samples following activation in the flux analysis (Figure 4A). This signifies that ME/CFS NK cells rely more heavily on long-chain fatty acids for energy during times of stress based on the larger shift in oxygen consumption rate (pmol/min) reported in ME/CFS cells compared to healthy control cells. In the ME/CFS cohort, we utilized two samples from the same ME/CFS patient, one collected before the patient began a therapy and one collected after the therapy had improved their symptoms, according to the clinical surveys. The sample collected after the treatment exhibited much greater fatty acid oxidation than the one collected when the patient was in poorer health (Figure 4A, circled). The increase implies that the patient’s NK cells were producing more energy after the treatment.

Following flow cytometric analysis, we observed significantly higher levels of exogenous fatty acid absorption in circulating ME/CFS NK cells compared with healthy control cells (Figure 4B). We also detected a similarly increased abundance of the supplied fatty acid in ME/CFS samples following activation (Figure 4B). When we assayed the MFI of CPT1a in these same samples, we saw a significantly higher abundance of this mitochondrial transporter in stimulated ME/CFS NK cells versus those from healthy controls while also seeing a comparably greater abundance of CPT1a in resting ME/CFS NK cells when compared to their resting healthy control counterparts (Figure 4D). These findings are consistent with the flux analysis, however, there appears to be some heterogeneity in the patient data for both the Bodipy and the CPT1a measurements. Nevertheless, we see a lower abundance of the cell membrane fatty acid transporter, CD36, in the same ME/CFS cell samples in circulation and after activation compared to healthy cells (Figure 4C). Thus, while ME/CFS NK cells exhibit increased levels of FAO in the mitochondria, CD36-mediated uptake into cells does not appear to factor in the differences between NK cells from cases vs. controls.

These combined findings, summarized in Table 2, show that NK cell fatty acid oxidation is higher in ME/CFS NK cells compared to their healthy counterparts; however, the cellular components we investigated that contribute to the upregulation of this pathway are not consistently greater in ME/CFS cells.

### 2.3. ME/CFS CD4+ T Cells Have Higher Levels of Fatty Acid Oxidation and Transporters Than Healthy Control Cells

We then sought to investigate the dependence of ME/CFS CD4+ T cells on fatty acids for energy. We calculated the change in OCR during the modified Mito Stress Test in circulating CD4+ T cells. Samples with sufficient circulating CD4+ and CD8+ T cells were selected to be assayed to accurately represent the state of these cells at the time that the individuals’ blood samples were drawn.

**Figure 5:**
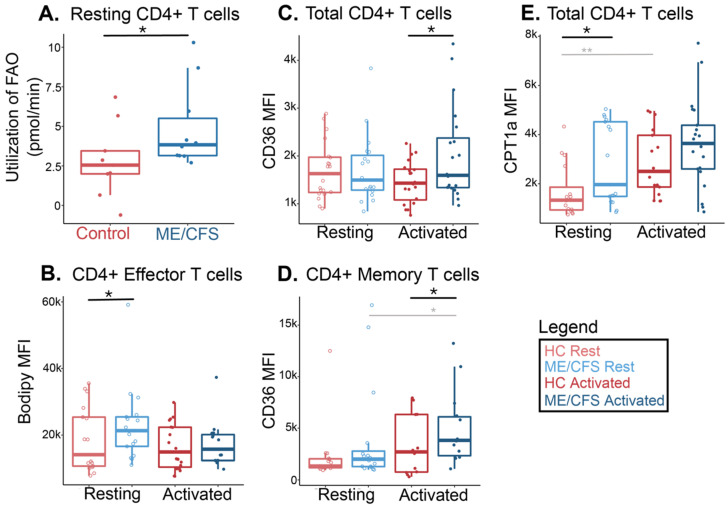
ME/CFS CD4+ T cells increased fatty acid oxidation compared to healthy controls. (**A**) Fatty acid oxidation measurements from a Seahorse extracellular flux analysis of circulating CD4+ T cells (*n* = 9 healthy control samples; *n* = 10 ME/CFS samples). Each dot represents the change in oxygen consumption rate (pmol/min) under high energy demands following the injection of etomoxir in one sample, with an average of 2–3 technical replicates. (**B**) Median fluorescence intensity of a supplied fatty acid (Bodipy) in circulating and activated CD4+ effector T cells (CD27-CD28-CD45RA+/-) (*n* = 16 healthy control samples at rest; *n* = 17 ME/CFS samples at rest; *n* = 19 healthy control samples after activation; *n* = 15 ME/CFS samples after activation, [*n* = 16/17/19/15]). (**C**) Median fluorescence intensity of a fatty acid cell membrane transporter, CD36, in total resting and activated CD4+ T cells (*n* = 19/19/19/20). (**D**) Median fluorescence intensity of a fatty acid cell membrane transporter, CD36, in resting and activated CD4+ memory T cells (CD27+CD28+CD45RA-) (*n* = 14/16/12/14). (**E**) Mean fluorescence intensity of a fatty acid mitochondrial transporter, CPT1a, in total resting and activated CD4+ T cells (*n* = 15/17/16/21). Box plots represent the median ± 25th and 75th quartiles. Whiskers represent 1.5× the interquartile ranges. Outliers are values outside the whisker range. Healthy control and ME/CFS data points are light (resting) or dark (activated) red or blue, respectively. For Seahorse data, * *p* < 0.05 by Wilcoxon rank-sum test. For flow cytometry data, * *p* < 0.05 and ** *p* < 0.01 by Kruskal–Wallis followed by Dunn’s test with FDR-based multiple testing correction. Gray lines and */** indicate a significant difference between ME/CFS and ME/CFS or healthy control and healthy control cohorts before and after stimulation.

To investigate the contribution of fatty acids in ME/CFS and healthy control CD4+ T cells, we used the Agilent Seahorse Xfp extracellular flux analyzer along with the aforementioned drug injection strategy (Figure 1B). CD4+ T cells from ME/CFS subjects had a greater change than controls’ cells in the oxygen consumption rate after injecting the fatty acid oxidation inhibitor etomoxir, indicating a greater contribution of this energy pathway compared to healthy control samples in a high-stress environment (Figure 5A). 

We next analyzed fatty acid utilization in ME/CFS CD4+ T cells by flow cytometry, both at rest and following overnight activation with anti-CD3/anti-CD28 beads and IL-2, as previously described [51]. In addition to the fatty acid markers, we used other cell surface markers to categorize T cells into naïve, effector, late effector memory, and memory cell populations (Figure 3, Supplementary Table S2). These experiments were carried out in an effort to locate any hidden sub-populations that could be using fatty acids excessively as fuels but are masked or unobserved in total cell populations. This type of cell gating will also enable us to identify populations driving any overall CD4+ T cell changes in fatty acid oxidation.

In all CD4+ T cell populations, we saw no significant differences in the MFI of the supplied fatty acid at rest or after activation between patients and healthy controls (Supplementary Figure S1A). However, in ME/CFS CD4+ effector T cells, which are identified as the helper or regulatory T cells of the immune system, we saw an increase in the absorption of the supplied fatty acid compared to the healthy control cells (Figure 5B). Similarly, we saw higher levels of this fatty acid abundance in ME/CFS naïve and late effector memory cells, both at rest and after activation (Supplementary Figure S1(Aii,iv)). There was high variability of Bodipy MFI in cells from all groups. We observed significantly higher amounts of fatty acid absorption upon activation in the memory T cell population of both cohorts, which is anticipated, particularly in older individuals (Supplementary Figure S2(Av)) [68].

When we assayed the cell surface fatty acid transporter, CD36, in CD4+ T cells, we saw an overall higher trend in all cell populations of activated ME/CFS samples compared to healthy controls (Figure 5C,D and Supplementary Figure S1B). Levels of CD36 were significantly different between cohorts in both memory and total CD4+ T cell populations, which could suggest that the ME/CFS memory subset is driving the difference in CD36 abundance that we see in the total population (Figure 5C,D).

Resting total CD4+ T cells showed a significantly greater abundance of CPT1a in ME/CFS samples versus healthy control samples (Figure 5E). An increase was also seen in the stimulated group at *p* = 0.16 (Figure 5E). There was no significant difference in CPT1a abundance between cohorts in the other populations. However, a slight trend was seen in a few instances that mirrored the pattern seen in total cells, where circulating ME/CFS naïve and effector cells had slightly higher levels of this transporter (Supplementary Figure S1(Cii,iii)). As expected, activation resulted in a significantly greater abundance of CPT1a in activated CD4+ memory T cells in both ME/CFS and healthy samples (Supplementary Figure S1(Cv)).

Overall, this data indicates that ME/CFS CD4+ T cells depend more on fatty acid oxidation than their healthy counterparts. We further summarized these findings in Table 3.

### 2.4. CD8+ T Cells Show Higher Levels of Fatty Acid Usage in ME/CFS Compared to Healthy Samples

**Figure 6:**
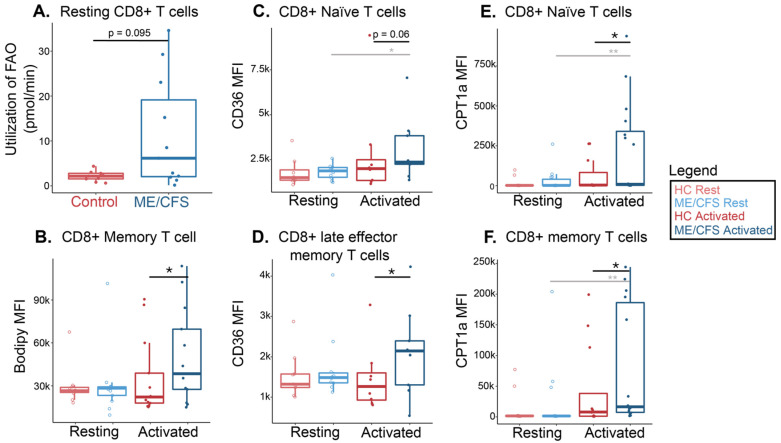
ME/CFS CD8+ T cells increased fatty acid oxidation compared to healthy controls. (**A**) Fatty acid oxidation measurements from a Seahorse extracellular flux analysis of circulating CD8+ T cells (*n* = 9 healthy control samples; *n* = 11 ME/CFS samples). Each dot represents the change in oxygen consumption rate (pmol/min) under high energy demands following the injection of etomoxir in one sample, with an average of 2–3 technical replicates. (**B**) Mean fluorescence intensity of a supplied fatty acid (Bodipy) in circulating and activated CD8+ memory T cells (CD27+CD28+CD45RA-) (*n* = 11 healthy control samples at rest; *n* = 13 ME/CFS samples at rest; *n* = 13 healthy control samples after activation; *n* = 13 ME/CFS samples after activation, [*n* = 11/13/13/13]). (**C**) Mean fluorescence intensity of a fatty acid cell membrane transporter, CD36, in resting and activated CD8+ naive T cells (CD27+CD28+CD45RA+) (*n* = 8/10/8/9). (**D**) Mean fluorescence intensity of a fatty acid cell membrane transporter, CD36, in resting and activated CD8+ late effector memory T cells (CD27-CD28-CD45RA+) (*n* = 9/10/8/9). (**E**) Mean fluorescence intensity of a fatty acid mitochondrial transporter, CPT1a, in resting and activated CD4+ naïve T cells (CD27+CD28+CD45RA+) (*n* = 11/13/12/14). (**F**) Mean fluorescence intensity of a fatty acid mitochondrial transporter, CPT1a, in resting and activated CD4+ memory T cells (CD27+CD28+CD45RA-) (*n* = 11/14/11/16). Box plots represent the median ± 25th and 75th quartiles. Whiskers represent 1.5× the interquartile ranges. Outliers are values outside the whisker range. Healthy control and ME/CFS data points are light (resting) or dark (activated) red or blue, respectively. For Seahorse data, * *p* < 0.05 by Wilcoxon rank-sum test. For flow cytometry data, * *p* < 0.05 and ** *p* < 0.01 by Kruskal–Wallis followed by Dunn’s test with FDR-based multiple testing correction. Gray lines and */** indicate a significant difference between ME/CFS and ME/CFS or healthy control and healthy control cohorts before and after stimulation.

To further explore fatty acid consumption in ME/CFS T cell populations, we determined the change in OCR during the revised Mito Stress Test in circulating ME/CFS and healthy control CD8+ T cells (Figure 1B). In CD8+ T cells, ME/CFS subjects had a substantially higher oxygen consumption rate change than healthy control cells following fatty acid oxidation inhibition (Figure 6A). This implies that ME/CFS CD8+ T cells absorb a higher quantity of fatty acids for oxidation during increased energy demands compared to healthy cells. The rate change is more widespread within the patient group, indicating that specific individuals with ME/CFS may utilize more fatty acids for energy in these cells compared to others. Indeed, there appear to be two populations of patients; one with FAO utilization similar to controls, while others have much greater rates (Figure 6A).

We next performed flow cytometric analysis on CD8+ T cells at rest and following overnight activation to characterize further fatty acid oxidation components and usage in T cell populations, as defined in Figure 2. Some samples had limited numbers of viable cells, resulting in low cell counts within smaller cell subsets, which thus were excluded from some population analyses. CD36 evaluation was limited in this cohort to ten patients and ten controls.

After supplying Bodipy to ME/CFS CD8+ memory T cells, we saw higher MFI levels of the absorbed fatty acid analog following activation compared to healthy controls (Figure 6B). There was a slightly higher MFI abundance of the fatty acid analog in the stimulated naïve ME/CFS CD8+ T cells versus healthy cells, but this was driven by about four outlying patient samples (Supplementary Figure S2(Aii)). Otherwise, there were no significant differences in Bodipy abundances in total, naïve, effector, or late effector memory cells between ME/CFS and healthy control CD8+ T cells (Supplementary Figure S2A). 

In stimulated cells, we found that the CD36 MFI was slightly higher in all ME/CFS CD8+ T cell populations compared to healthy controls, but this difference was only significant in late effector memory T cells (Figure 6C,D, Supplementary Figure S2(Bi,iii,v)). Naïve CD8+ T cells also had a significantly greater abundance of CD36 between rest and stimulation in ME/CFS samples, which was not seen in healthy controls (Figure 6C). This pattern also appeared in total, effector, late effector memory, and memory ME/CFS CD8+ T cell populations, though it was not as substantial (Figure 6D, Supplementary Figure S2(Bi,iii,v).

After overnight stimulation, naïve and memory CD8+ T cell subsets showed greater CPT1a abundance in ME/CFS compared to healthy cells (Figure 6E,F). We also detected higher levels of CPT1a between rest and activation in ME/CFS samples within these two populations, which was not seen in healthy control samples (Figure 6E,F). The MFI levels of the mitochondrial transporter, CPT1a, were not significantly different in any resting CD8+ T cell populations between the two cohorts (Supplementary Figure S2(Ci,iii,iv) and Figure 6E,F).

These findings indicate that, specifically in activated samples, ME/CFS CD8+ T cell subpopulations exhibit higher levels of important fatty acid oxidation proteins and utilize greater amounts of fatty acids when under stress relative to healthy CD8+ T cells (Table 4). There is also a wider range of values in this dataset, particularly in the stimulated ME/CFS cohort, which could suggest that the immune cells of certain participants are favoring this energy pathway during a simulated immune challenge more than cells of other subjects.

### 2.5. CD4+ T Cell Fatty Acid Oxidation Data Correlates with Participant Survey Data

The above metabolic analyses allowed us to identify distinct bioenergetic characteristics in ME/CFS lymphocytes. However, we also observed some instances of heterogeneity in the ME/CFS cohort’s FAO metabolic profile compared to the healthy controls, leading us to question whether subject phenotype may be influencing our results. Thus, we obtained survey data and demographics from each participant, including records for their age, sex, disease duration, and illness severity measurements (Table 1 and Supplementary Table S1). We used Spearman’s correlation test with FDR adjustment for multiple testing. An FDR-adjusted *p* value (*q*-value) of less than 0.05 was considered a significant correlation. We also performed a Pearson’s product–moment test for correlations between sex, where a *p*-value of less than 0.05 was considered significant.

**Figure 7:**
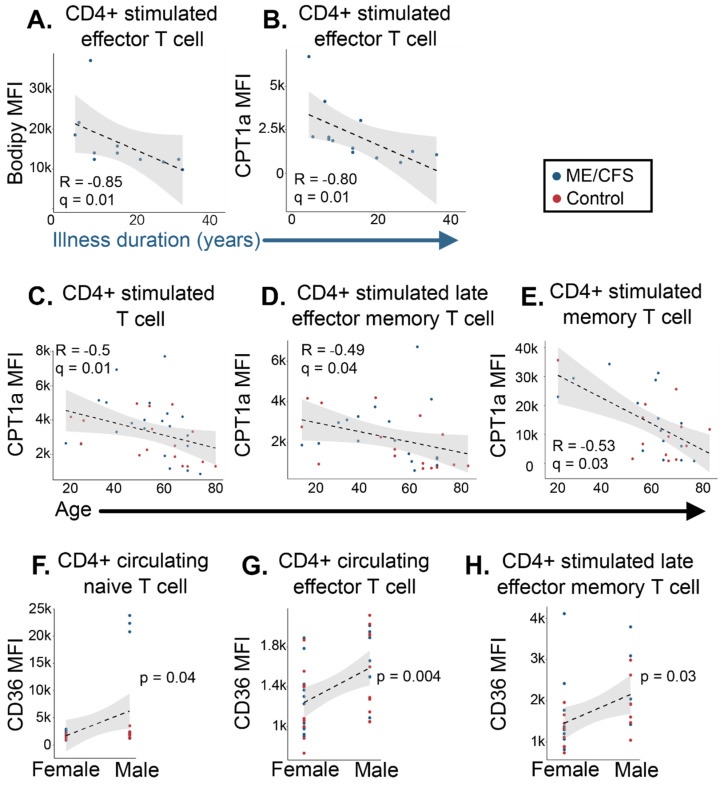
CD4+ T cell fatty acid oxidation markers correlate with survey data in patients with ME/CFS and healthy controls. Significant correlations between subject information and CD4+ T cell fatty acid oxidation data. Plots of correlations between (**A**) stimulated effector T cell Bodipy (*n* = 11) and (**B**) CPT1a (*n* = 13) MFI vs. duration of illness; (**C**) stimulated total (*n* = 16 healthy control samples, *n* = 20 ME/CFS samples [*n* = 16/20]), (**D**) late effector memory (*n* = 16/17), and (**E**) memory (*n* = 13/15) CPT1a MFI vs. age, and (**F**) circulating naïve (*n* = 12 healthy control samples, *n* = 19 ME/CFS samples, and *n* = 17 females, *n* = 14 males [*n* = 12/19, 17/14]), (**G**) circulating effector (*n* = 19/20, 22/17), and (**H**) stimulated late effector memory (*n* = 16/14, 18/12) CD36 MFI vs. sex. For correlations (**A**–**E**), a Spearman’s correlation test with FDR-based multiple testing correction was performed, where a q value of less than 0.05 was considered significant. For correlation testing between sex (**F**–**H**), a Pearson’s product-moment correlation was used, where a *p*-value of less than 0.05 was considered significant.

We found no significant correlations between NK cell nor CD8+ T cell fatty acid oxidation parameters and symptom survey data in patients with ME/CFS or healthy participants. We did, however, discover a number of significant correlations between CD4+ T cell fatty acid data and participant information. For instance, we found that in activated CD4+ effector T cells, levels of the supplied fatty acid analog, Bodipy 558/ 568, and the mitochondrial membrane fatty acid transporter, CPT1a, were both negatively correlated with the duration of illness for the patient cohort (Figure 7A,B). CPT1a abundance also correlated with subject’s age—we found a negative correlation between all participants’ age (both ME/CFS and healthy samples) and levels of CPT1a MFI in stimulated CD4+ total, late effector memory, and memory T cell populations (Figure 7C–E). Interestingly, we observed three significant correlations between sex and levels of CD36 in total samples. Males exhibited a higher abundance of CD36 in circulating CD4+ naïve and effector T cell populations compared to the female cohort (Figure 7F,G). In the naive CD4+ population, a few of the male patient group participants seemed to drive this difference compared to the control cohort (Figure 7F). The cell membrane fatty acid transporter levels were also higher in the stimulated CD4+ late effector memory T cell male cohort versus the female group (Figure 7H). As males and females were distributed throughout all cell type cohorts, it is somewhat surprising that these correlations exist only within the context of CD4+ T cells. Combined, this data further highlights the immune dysregulation seen in ME/CFS and perhaps illustrates the stratification of irregularities in metabolism that occur on the basis of age, sex, and the length of an illness.

## 3. Discussion

Previous studies have linked lymphocyte dysfunction to ME/CFS, particularly in T and NK cell populations. Immune system involvement in ME/CFS is comprised of changes in cytokines, a diminished cytotoxic immune response, poorer proliferative capabilities, and patient symptoms, such as inflammation and flu-like instances, that reflect immune impairment [3]. Meanwhile, plasma metabolomic studies in ME/CFS consistently report altered lipid abundances in ME/CFS [28,30,31,32,33,34,35]. Studies from our lab have previously reported an increased abundance of two carnitine compounds and significant differences in multiple fatty acid and lipid pathways through pathway and statistical enrichment analyses of ME/CFS plasma metabolites [33,34,35]. These investigations can only infer cellular function based on blood metabolite levels; however, the metabolomic anomalies found in ME/CFS patients’ plasma could come from many cell and tissue types. Prior immunometabolism studies in total PBMCs cannot determine whether particular cell types have a differential contribution to the overall view of the metabolic pathways. The novelty of our findings comes from teasing apart the isolated immune cell populations to uncover the direct influences of irregular metabolic remodeling during activation that could inform specific cell function, or rather, dysfunction in ME/CFS. Deviations from appropriate metabolic pathways in immune cells can facilitate disease onset and management, and our results confirm a shift in metabolic pathways in isolated ME/CFS lymphocytes.

### 3.1. Study Population and Correlations between Participant Information and FAO Data

Our study population was an accurate reflection of the health status of the ME/CFS community, as seen in the survey data collected. The ME/CFS group clearly demonstrated greater disability than the healthy cohort. Age distribution between the cohorts was balanced, and the patient group contained a higher number of female patients than males in all cell types, which is a commonly reported statistic in ME/CFS studies [69]. The illness duration of our patient population varied considerably from a year to 54 years, with the average length representing mainly those with a longer duration of the disease. This was not unexpected given that the typical time from the onset of symptoms to a diagnosis is nearly 7 years [3]. 

It was intriguing to see the negative correlation between a patient’s illness duration and measurements of fatty acid utilization and metabolic markers. This would imply that individuals who had been sick longer utilized fatty acid oxidation less in CD4+ effector T cells than those who acquired the disease more recently. This result is difficult to interpret, as there is no research on comparative FAO levels in the immune cells of people who have been ill for decades. Studies on “long-term” antigen exposure and metabolism typically occur in cell lines and analyze data gathered a week from exposure up to a month—this does not accurately represent our patient cohort since many of them have been living with the disease for decades. This correlation could suggest that those with ME/CFS who have had the condition for a longer time rely on fatty acids less for ATP production than those in earlier stages of the illness. Combining this knowledge with our earlier research, which also revealed lower glycolysis in this cell type, suggests that longer-suffering patients may simply be less adept at producing ATP in general [51].

The negative correlations that we see between age and CD4+ T cell CPT1a abundance point to a decreased reliance on fatty acids for energy during an immune challenge as a person ages. While studies have examined this phenomenon, they seem to contradict each other and may not completely mirror our specific population and cell environment. One study reported that CD4+ T cells from individuals 65 and older expressed higher CPT1a levels compared to those who were no older than 35 [68]. A different study in young vs. aged mice found an increase in fatty acid oxidation metabolites in the older cohort’s CD4+ T cells using mass spectrometry [70]. Our cohort differs from these two studies in that our correlation is significant for total samples, not discriminating between ME/CFS and healthy subjects nor excluding individuals in our cohort who were between 35 and 65 years old. Moreover, the amount of time and type of stimulating agents used in our cell activation tactics differed. Cell isolation strategies were also dissimilar; while we isolated CD4+ T cells with magnetic beads, one study used negative selection density-based antibody separation, and the other used FACS sorting, which can alter cells’ redox and metabolic states [71].

The sex differences we noticed in naïve, effector, and late effector memory CD4+ T cell fatty acid transporter abundances are not entirely surprising. Evidence of disparities based on sex has been reported in ME/CFS plasma metabolites and even antibodies [30,35,72]. Outside of the context of ME/CFS, researchers have broadly noticed immune system discrepancies between the sexes [10]. For example, the female innate and adaptive immune response is typically more extensive than the male response [73]. Specifically, in CD4+ T cell populations, female cells show a greater proportion, activation, and proliferative capability than male cells. Research also suggests that females are better protected during an immune challenge because of their ability to produce higher levels of helper T cells than males. Moreover, there seems to be a preference in cytokine production within this cell type based on sex—in one study, naïve CD4+ T cells from females produced more IFN-γ upon stimulation, while the immune system in males opted towards IL17 under the same conditions [74]. IL17 production requires fatty acid uptake, so our observation of higher CD36 levels in male effector and stimulated late effector memory cells could be related to this cytokine preference [75]. While our data shows three CD4+ T cell populations with higher levels of CD36 in males versus the female cohort, it should be noted that the difference in naïve cells seems to be driven by three individuals in the patient population. The other two cell population correlations are more evenly distributed between patients and healthy controls. These correlations may also be consistent with the observations that females are more responsive to infections by suggesting that males are less able to switch their metabolic state from FAO to the more glycolytic or glutamine-dependent pathways during stimulation that allows T cells to synthesize crucial byproducts needed for proliferation and effector function [76].

### 3.2. Effects of Lipid Metabolism Abnormalities in ME/CFS NK Cells

Our study of fatty acid oxidation revealed that ME/CFS NK cells increased their use of this pathway during stress, absorbed more of the supplied fatty acid, and had higher levels of CPT1a in circulation. These metabolic differences were not overcome after stimulatory factors were added. Conversely, patients had lower levels of the cell membrane lipid transporter, CD36, in both resting and activated states. The finding that FAO is used more often within these cells while CD36 is less abundant may be attributed to CD36’s dual functions, not only as a fatty acid transporter but as a regulator of intracellular fatty acid homeostasis. The downregulation of CD36 in environments with high amounts of surrounding lipids can reduce cellular lipid overaccumulation by promoting the oxidation of fatty acids already present in the cell, according to research on CD36 in hepatocytes. This regulatory mechanism has been observed in fatty acid liver disease and metabolic syndromes [77]. Previous metabolomic findings support this theory, as they report high levels of certain fatty acids in ME/CFS plasma and hypothesize that it could be a sign of hyperlipidemia or a deficiency in liver activity [33].

High levels of fatty acids in and surrounding these cells—and the subsequent increased fatty acid oxidation—could also explain a commonly cited finding in ME/CFS literature: decreased NK cell cytotoxicity [19,20,21,22,23,24]. Lipid accumulation has been shown to suppress mTOR activity and block cytotoxic machinery necessary for NK cell functionality [55,78,79]. If excess free fatty acids block the cellular components necessary for effector functions, ME/CFS NK cells will have a lower cytotoxic response than healthy individuals. This, in turn, will lead to a lower capability of the innate immune system to defend against virally infected cells [80]. In practice, these findings suggest that targeting or rewiring fatty acid metabolism through multiple additive mechanisms could improve NK cell function in individuals with ME/CFS [55].

### 3.3. Implications of Altered Lipid Metabolism in ME/CFS T Cells

Our drug injection strategy for flux analysis mimicked a state of inflammation by injecting cells with FCCP, which results in the quick oxidation of sugars, fats, and amino acids to meet this high-energy request. In the human body, these nutrient, oxygen, and ATP demands occur to carry out phagocytosis and microbial killing. By injecting etomoxir, we were able to determine how much long-chain fatty acids are contributing to the cell’s ability to meet this metabolic challenge. In both circulating CD4+ and CD8+ T cells from patients with ME/CFS, we found increases in fatty acid oxidation under high energetic demands for ATP production compared to healthy controls. Previous metabolic assays in isolated CD4+ T cells, CD8+ T cells, and total PBMCS reported decreased glycolysis in ME/CFS subjects [44,51]. This suggests that fatty acids may be one of the fuels used by T cells to compensate for the lower glycolytic rates observed in this disease.

In CD4+ T cells, flow cytometric analysis of other fatty acid metabolic components seemed to concur with the live cell metabolic rate assays. Total CD4+ T cells demonstrated higher abundances of both cell surface and mitochondrial fatty acid transporters. CPT1a abundance was greater at rest in ME/CFS, while both transporters had higher levels after stimulation, significantly so for CD36. Upregulation of these two metabolic enzymes in CD4+ T cells can promote Treg production [81]. One study suggests that it is the combination of increased FAO and glycolysis that allows Tregs to proliferate in stressful environments, but ME/CFS subjects do not have a normal glycolytic capacity in these cells, which could be limiting the prevalence of this effector cell [82]. This highlights the importance of using combined approaches when considering metabolic components for therapeutic options—altered levels of other nutrients such as amino acids and glucose in ME/CFS immune cells should be considered and investigated to create treatment avenues in ME/CFS and achieve proper cellular function.

The subpopulations of CD4+ T cells that showed significant or substantial trends in this data included effector and memory subsets. Circulating effector CD4+ T cells in ME/CFS had a higher abundance of the supplied fatty acid than healthy control cells, indicating that the helper or regulatory T cells in ME/CFS were taking up higher levels of exogenous fatty acids. In other diseases with rampant inflammation, like multiple sclerosis, increased fatty acid uptake enhances Th1 and Th17 differentiation, causing an exacerbated disease state [83]. Memory CD4+ T cells also showed a greater abundance of CD36 in activated ME/CFS samples compared to healthy samples. While FAO reliance is typically greater in memory T cells, the fact that we are seeing even higher abundances of CD36 in the stimulated ME/CFS population could indicate that these cells have a unique requirement for acquiring exogenous fatty acids to fuel mitochondrial respiration [58]. One study of effector memory CD4+ T cells in tumors demonstrated that these cells limit their ability to metabolize fatty acids to retain T cell function in nutrient-limited microenvironments [54]. Likewise, older CD4+ memory T cells that increased FAO during proliferation led to a reduced capability of the cells to survive [68]. If ME/CFS memory CD4+ T cells are unable to remodel their metabolism to decrease fatty acid dependency, these findings suggest that they could have depleted effector functions during high energy demands.

While there were no significant differences found in total ME/CFS CD8+ T cell fatty acid data compared to healthy control cells, we began to see alterations once we analyzed the different cell types within this population. In ME/CFS naïve, memory, and late effector memory CD8+ T cells, we saw higher long-chain fatty acid transporter abundances in stimulated conditions, suggesting that during an immune challenge, these cell populations rely more on fatty acids for fuel than healthy control cells. Stimulated ME/CFS memory T cells also revealed a higher abundance of the supplied fatty acid compared to healthy memory cells. While generally, naïve and memory cells depend on FAO and glycolysis to sustain basic cellular function, support migration, and, in memory cells, persist for long periods of time, this greater FAO dependency could be indicative of alternative fuel usage in T cells compensating for the defects in glycolysis [51,84]. The naïve FAO increase could indicate an inability or delay in metabolic remodeling for subsequent effector differentiation and function—decreases in CD8+ T cell proliferative response and effector/memory populations have been reported in ME/CFS subjects [16]. There are studies that propose higher levels of long-chain fatty acid oxidation in memory CD8+ T cells supply additional energy needed for sustenance and proliferation after the removal of antigenic stimulation, but this may not reflect environments in vivo or the particular conditions in and around ME/CFS cells, since other important energy-producing metabolic pathways are disrupted in this disease [43,44,45,46,47,51,84]. However, notable interventions to overcome altered metabolic conditions aim to stimulate mitochondrial activity or deliver metabolic modulators that favor the induction of memory T cell phenotypes. These treatments can precondition T cells to maintain certain metabolic pathways and enhance cytotoxic function before adoptive transfer to a patient [85]. By looking at these subsets independently, we can see the contributing cell types that are perhaps trying to compensate for the decrease in glycolysis by increasing their fatty acid energy usage, but even these higher rates may not be enough energy to support proper cell function in ME/CFS [86].

Metabolic features and immune cell dysfunction in ME/CFS T cells, such as decreased mitochondrial membrane potential, glycolytic impairments, lower granzyme A/perforin production, and now higher levels of fatty acid oxidation, are also consistent with an exhausted T cell state, common in chronic viral infections and cancer [23,51,53,87]. These characteristics are found in individuals experiencing long-term exposure to antigens, subsequently suppressing T cell survival, proliferation, and cytokine production [52,53]. Various groups have studied T cell exhaustion extensively, and abnormal metabolic regulation is not a consequence of this phenomenon but, rather, drives this immune cell state. Inhibition of CPT1a significantly reduced mitochondrial respiration in early exhausted T cells, and the study suggested that fatty acid usage was a survival tool instigated in glucose-limiting environments but was perhaps still not sufficient to support a robust immune response [86]. Blocking inhibitory receptors such as PD-1, CTLA-4 or Lag-3 have reversed this exhausted state in clinical trials of T cells in cancer [88]. Additionally, metformin has been utilized in HIV-induced T cell exhaustion treatment to increase glucose import and subsequent cellular energy production [89]. Meanwhile, knocking out CD36 in Tregs of a hypoxic tumor microenvironment allowed lymphocytes to kill target cells while sustaining homeostasis, pointing to the importance of considering metabolic components like these in therapeutic treatments [90].

### 3.4. Strengths and Limitations 

This study’s main strengths lie in the patient population data collected and the sensitivity of examining several distinct subpopulations in these cell samples in circulation and following stimulation. By separating the PBMCs into different cell types prior to metabolic analyses, we can determine the drivers and hidden subtypes displaying altered bioenergetics in lymphocytes. Moreover, using a comprehensive flow cytometric panel, we can further characterize T cell populations contributing to dysfunctional metabolic states. This direct analysis of distinct cells provides more information about how and why immune cells are not functioning properly in this disease. Additionally, it has the potential to inform researchers about more focused therapy ideas that might target certain cells and their metabolic abnormalities.

A limitation for some of our experiments was the small sample size—variability in cell number and sample quality sometimes limited our data analysis. Future studies should consider distinguishing T cell populations further into Tregs, Th1, Th2, and other helper cells when analyzing metabolic characteristics—examining the metabolic states of these cell types could apprise us of the functional changes observed in the ME/CFS immune response since certain cell populations rely on different metabolic pathways to function. For example, CD4+ helper cells, such as Th1, Th2, and Th17, depend on glycolysis for proper effector function. Meanwhile, Tregs, which balance and suppress the immune response, rely more heavily on fatty acids to fuel the cell’s anti-inflammatory response [41,42]. Our study was also limited in the type of fatty acids investigated. Further investigation into the potential of short, medium, and very long chain fatty acids to be oxidized by T cells would be of interest in this disease. Previously, evidence for use of medium-chain fatty acids to sustain T cell proliferation during activation has been observed, so characterizing the use of these molecules individually in ME/CFS could be informative [84]. Another important area of study for these cell types will be to perform functional assays during metabolic perturbations to test the theory that there is a suppressed immune response as a result of an altered metabolic state in this disease.

## 4. Materials and Methods

### 4.1. Study Population

The majority of study participants were recruited by Simmaron Research (Incline Village, NV, USA), where the subjects with ME/CFS were established patients of Dr. Daniel Peterson. This cohort’s healthy controls and patients with ME/CFS completed approved questionnaires. These included an SF-36 survey [57], a Bell Activity Scale, a specific symptom inventory, and general questions pertaining to symptoms, comorbidities, and a family health history of each subject. Additional study participants were individuals involved in the NIH-funded Cornell Collaborative ME/CFS Center at Ithaca College, Weill Cornell Medicine, and EVMED or visited Ithaca College to seek cardiopulmonary exercise testing. All subjects with ME/CFS were in accordance with the Canadian Consensus Criteria for ME/CFS diagnosis [91]. Sample sizes and further survey information can be found in Table 1 and Supplementary Table S1.

### 4.2. Sample Collection and Blood Processing

Whole blood from each subject was collected into EDTA tubes for a total of 80 mls and processed within 1–2 h into aliquots of whole blood, plasma, and PBMCs, as previously described [51]. A visualization of this protocol can be found in Figure 1A. EDTA tubes were spun at 500× *g* for 5 min, and plasma was separated and stored at −80 °C. Blood was diluted 1:2 in PBS and layered over Histopaque 1077 (Sigma-Aldrich, St. Louis, MO, USA) in 50-mL SepMate tubes (STEMCELL Technologies, Vancouver, BC, Canada). SepMate tubes were spun at 1200× *g* for 10 min, excess plasma was removed, and cells were put into sterile 50-mL conical tubes. Platelets were depleted from the cells by first washing them in PBS at 120× *g*, and then again at 300× *g* for 5 min. PBMCs were then resuspended in freezing medium (60% RPMI 1640, 30% heat-inactivated FBS, 10% DMSO) and stored at −80 in isopropanol-containing freezing containers to slow down freezing. A few PBMC aliquots were transported to the Hanson lab overnight on dry ice from New York City and Los Angeles. Liquid nitrogen was used to store PBMCs for long-term use.

### 4.3. Immune Cell Isolation

Specific immune cell subsets (CD56+, CD8+, and CD4+) were isolated using STEMCELL EasySep kits on a STEMCELL EasyEights magnet, as seen in Figure 1A and explained in Mandarano et al. [51]. Briefly, PBMCs were thawed in a 37 °C water bath and washed in RPMI 1640. To remove any leftover cell clumps, PBMCs were strained through a 37-μm cell strainer after being treated with 10 mg/mL DNase I for 10 min at room temperature. After a second wash, cells were isolated following manufacturer’s instructions using the EasySep Human CD19 Positive Selection II Kit, the EasySep Human CD56 Positive Selection II Kit, the EasySep Human CD8 Positive Selection II Kit, and the EasySep Human CD4+ T Cell Isolation Kit, sequentially. Isolated cells were subsequently frozen and stored using the freezing medium and protocol explained above.

### 4.4. Agilent Seahorse Extracellular Flux Analysis

Extracellular flux experiments were conducted on a Seahorse XFp (Agilent Technologies, Santa Clara, CA, USA). Isolated immune cells for resting assays (CD4+ and CD8+ T cells, described in Section 4.3) were thawed in a 37 °C water bath on the day of the experiment and washed in Seahorse assay media (Seahorse XF RPMI Medium pH 7.4, 10 mM Seahorse XF glucose, 2 mM Seahorse XF L-glutamine, 1 mM Seahorse XF pyruvate). Cells were counted on a Bio-Rad TC20 with trypan blue to measure the viability and count prior to seeding in triplicate on a Seahorse XFp Cell Culture Microplate treated with Cell-Tak (22.4 mg/mL) (Corning, ThermoFisher Scientific, Waltham, MA, USA). The plate was centrifuged at 300× *g* for 1 min with no brake to adhere cells. Sterile water was added to each moat in the plate and assay media was added to the experimental cells to raise the volume to 180 μL per well. The plate was spun one final time at 300× *g* for 1 min with no brake to further confirm that cells would adhere to the bottom of the plate. Assays were run using a Seahorse Mito Stress Kit, with an added injection of the CPT1a inhibitor etomoxir [58,59,60,61]. A graphical representation of this drug injection strategy can be seen in Figure 1B. Drugs were injected at the following final concentrations: 1 μM oligomycin, 1 μM FCCP, 5 μM etomoxir, and 0.5 μM rotenone/antimycin A. For NK cell activation, isolated immune cells were cultured in media (89% RPMI-1640 with L-glutamine, 10% heat-inactivated FBS, and 1% sodium pyruvate) overnight prior to the assay, with the addition of stimulating agents, IL-15 (100 ng/mL, Life Technologies, Carlsbad, CA, USA) and IL-12 (30 ng/mL, BioVision, Milpitas, CA, USA). 

Flux analysis data were first normalized based on cell-seeding density per 100,000 viable cells using the Seahorse Analytics web-based Software (v.1.0.0-524). Data was further analyzed in RStudio. The oxygen consumption rate (OCR) was generated by this assay for each time point following a drug injection, and the change in OCR (pmol/min) was calculated by subtracting the lowest OCR measurement following the etomoxir injection from the highest OCR measurement following the injection of FCCP. The average of this value was calculated within replicates for each sample. Data were utilized only if at least 2 replicates provided reliable, quality data for a given variable. Basal respiration and maximal respiration were required to be positive (above nonmitochondrial respiration levels), while ATP production levels could not be greater than the total basal respiration. Therefore, the change in OCR measurements is an average of 2–3 technical replicates. 

### 4.5. Flow Cytometry

Flow cytometric analysis was performed on a ThermoFisher Attune NxT Analyzer at the Cornell Biotechnology Resource Center. Each cell type was stained with a specific cocktail of antibodies at rest and following stimulation (Supplemental Table S2). T cells were stimulated with STEMCELL Technologies’ reagents Immunocult (anti-CD3/anti-CD28, 25 μL/mL) and IL-2 (80 U/mL). NK cells were stimulated as described above. All cells were thawed and cultured overnight in media (89% RPMI-1640 with L-glutamine, 10% heat-inactivated FBS, and 1% sodium pyruvate) prior to staining. Antibodies used for staining can be found in Supplementary Table S2. Cells were moved to 15-mL conical tubes and first incubated with BODIPY 558/568 C12 (8.5 μM) for 20 min at 37°C. After 2 washes with PBS, cells were then incubated with surface stains listed in Table S2 for 20 min on ice in the dark. Cells were then washed and resuspended following BD Bioscience’s Fixation/Permeabilization kit protocol. Cells were then washed with BD Bioscience’s Perm/Wash buffer, resuspended in 100 μL with 1 μL of CPT1a, and incubated on ice for 30 min. Finally, after washing, the cells were incubated with a ThermoFisher Alexa Fluor 488 secondary antibody for 20 min on ice in the dark. Cells were washed, resuspended in 200 μL of BD Biosciences Stain Buffer (BSA), and moved to 5-mL round-bottom polystyrene tubes.

Flow cytometric analysis was conducted immediately following the staining protocol. Data analysis was conducted using FlowJo Software (v.10.8.1, Beckman Coulter, Brea, CA, USA). The gating strategy can be found in Figure 1C. Briefly, all cells were first gated by size, granularity, and viability. NK cells were then gated to include CD56+CD16+, CD56+CD16-, and CD56-CD16+ cells. CD8+ T cells were gated for naïve (CD27+CD28+CD45RA+), effector (CD27-CD28-CD45RA+/-), late effector memory (CD27-CD28-CD45RA+), and memory (CD27+CD28+CD45RA-) cell populations, based on previous studies using these markers for cell population identification [65,66,67]. The same populations were identified in CD4+ T cells. Within each of these populations and in total CD8+ and CD4+ T cells, the MFI (mean or median fluorescence intensity) of BODIPY 558/568 C12, CPT1a, and CD36 were calculated. Fluorescence Minus One (FMOs) was used to call positive signals. Samples were excluded if the cell count within a cell population was less than 20. ThermoFisher UltraComp eBeads Plus were used for running single stain compensations to create a compensation matrix for this assay.

### 4.6. Confocal Microscopy

Confocal microscopy was performed on immune cells using a Zeiss LSM710 at the Cornell Biotechnology Resource Center. Cells were incubated at 37 °C overnight at rest or after adding activating agents, as mentioned previously. Cells were incubated with Hoechst 33342 (2 μg/mL) and BODIPY 558/568 C12 (8.5 μM) for 20 min at 37 °C, washed twice, and resuspended with BD Bioscience’s Perm/Wash buffer. The cells were incubated for 20 min on ice and washed with BD Bioscience’s Perm/Wash buffer. Cells were then resuspended in 100 μL with 1 μL of CPT1a and incubated on ice for 30 min. Next, cells were washed and incubated with a ThermoFisher Alexa Fluor 488 secondary antibody for 20 min on ice in the dark. Cells were then washed in RPMI 1640 media without FBS and moved to a glass-bottom 24-well MatTek plate coated with 22.4 mg/mL Cell-Tak. The plate was spun down at 300× *g* for 1 min with no brake. Images were captured immediately after staining by using a ×63 oil immersion objective with 12-bit depth, bidirectional scanning, and a 3.6× magnification. The images represent a single image or focus-stacked images. Maximum intensity projections were produced from the focus stacks by utilizing Zen 2.5 software (Zeiss, Oberkochen, Germany). 

### 4.7. Statistics

RStudio and Microsoft Excel were used to conduct all statistical analyses. For survey data in Table 1 and Supplementary Table S1, survey responses between ME/CFS and control subjects were compared with pairwise statistical testing using a Wilcoxon rank-sum test. For the Seahorse extracellular flux analysis, pairwise comparisons between healthy control and ME/CFS cells were also done using a Wilcoxon rank-sum test. For comparisons of 4 groups within the flow cytometry assays (ME/CFS cells at rest, control cells at rest, activated ME/CFS cells, activated control cells), statistical testing was conducted via a Kruskal–Wallis test followed by Dunn’s test with FDR-based multiple testing correction. Spearman’s correlation tests were performed on all data for correlation testing, followed by an FDR-based multiple testing correction. A *q*-value of less than 0.05 was considered significant. For correlation testing between sex, a Pearson’s product–moment correlation was performed, where a *p*-value of 0.05 or less was considered significant.

## 5. Conclusions

Our findings support the theory of a consistently altered bioenergetic state in ME/CFS immune cells, specific to certain immune cell types that rely on fatty acids more heavily than healthy control subjects. This study detected increases in fatty acid oxidation in CD4+ T cells, CD8+ T cells, and NK cells at varying degrees, with CD4+ T cell fatty acid utilization correlating with ME/CFS illness duration. This data indicates that ME/CFS NK cell dysfunction could be in part due to greater levels of lipid accumulation and subsequent fatty acid oxidation. We also propose that the combined metabolic profile of ME/CFS T cells suggests an exhausted T cell state, resulting in reduced effector functions that may contribute to ME/CFS symptom presentation.

## Figures and Tables

**Table 1:** Study population demographics and survey data by cell type analyzed.

Cell Type			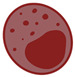 NK Cells			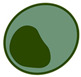 CD4+ T Cells			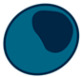 CD8+ T Cells	
			ME/CFS	Healthy Controls		ME/CFS	Healthy Controls		ME/CFS	Healthy Controls
*n*			28	26		29	26		44	39
Age			52.3 ± 13.0	52.4 ± 12.5		50.4 ± 15.4	50.0 ± 17.2		49.6 ± 15.5	47.9 ± 14.2
Illness Duration (years)			20.0 ± 10.8 (*n* = 21) (Range: 1–36 yrs)			16.0 ± 11.4 (*n* = 21) (Range: 1–36 yrs)			17.2 ± 12.0 (*n* = 29) (Range: 1–54 yrs)	
Bell Activity Scale			30.6 ± 16.2 (*n* = 18)	98.1± 4.0 (*n* = 16)		37.0 ± 17.7 (*n* = 18)	96.5 ± 6.1 (*n* = 17)		32.7 ± 14.1 (*n* = 24)	98.7 ± 3.5 (*n* = 23)
Sex	Male		11	8		12	16		15	16
	Female		17	18		27	11		29	21
Onset type	Gradual		7	-		8	-		12	-
	Sudden		11	-		11	-		13	-
	Unknown		10	-		10	-		19	-
SF-36	*n*		21	15		21	16		28	22
Physical component score			22.6 ± 7.3	51.8 ± 7.1		25.2 ± 8.6	51.0 ± 8.2		21.7 ± 7.5	54.2 ± 7.1
Mental component score			42.5 ± 13.5	55.1 ± 8.7		43.3 ± 11.7	51.5 ± 8.8		43.1 ± 12.0	51.1 ± 9.1

**Table 2:** Summary of results concerning FAO in ME/CFS vs. control NK cells.

Cell Type	Assay or Marker	Trend in ME/CFS *	*p*-Value
Circulating Total NK cells	Bodipy	Higher	0.007
	CD36	Lower	0.03
	CPT1a	Higher	0.07
Activated Total NK cells	FAO flux analysis	Higher	0.04
	Bodipy	Higher	0.07
	CD36	Lower	0.004
	CPT1a	Higher	0.01

* Green font indicates a higher trend of the related marker in the ME/CFS cohort, while a red font indicates a lower marker trend in the ME/CFS cohort.

**Table 3:** Summary of results concerning FAO in ME/CFS vs. control CD4+ T cells.

Cell Type		Assay or Marker	Trend in ME/CFS *	*p*-Value
Circulating CD4+ T cells	Total cells	FAO flux analysis	Higher	0.04
		CD36	No difference	0.47
		CPT1a	Higher	0.01
	Effector cells	Bodipy	Higher	0.05
	Memory cells	CD36	No difference	0.15
Activated CD4+ T cells	Total cells	CD36	Higher	0.03
		CPT1a	No difference	0.16
	Effector cells	Bodipy	No difference	0.32
	Memory cells	CD36	Higher	0.03

* Green font indicates a higher trend of the related marker in the ME/CFS cohort.

**Table 4:** Summary of results concerning FAO in ME/CFS vs. control CD8+ T cells.

Cell Type		Assay or Marker	Trend in ME/CFS *	*p*-Value
Circulating CD8+ T cells	Total cells	FAO flux analysis	Higher	0.095
	Naïve cells	CD36	No difference	0.34
		CPT1a	No difference	0.32
	Late Effector Memory cells	CD36	No difference	0.27
	Memory Cells	Bodipy	No difference	0.48
		CPT1a	No difference	0.49
Activated CD8+ T cells	Naïve cells	CD36	Higher	0.06
		CPT1a	Higher	0.02
	Late Effector Memory cells	CD36	Higher	0.04
	Memory Cells	Bodipy	Higher	0.04
		CPT1a	Higher	0.04

* Green font indicates a higher trend of the related marker in the ME/CFS cohort.

## Data Availability

All relevant summary data are within this article. Additional raw and representative data contributing to the summary figures are included in the Supplementary Figures.

## Supplementary Materials

The following supporting information can be downloaded at: https://www.mdpi.com/article/10.3390/ijms24032010/s1.
