# Supplementary material for: Altered Fatty Acid Oxidation in Lymphocyte Populations of Myalgic Encephalomyelitis/Chronic Fatigue Syndrome
Source: Int J Mol Sci. 2023 Jan 19;24(3):2010. doi: 10.3390/ijms24032010 (PMC9916395; doi:10.3390/ijms24032010)
Supplement: Supplementary file 1 [file ijms-24-02010-s001.zip › ijms-2144956-supplementary.pdf]

# Altered Fatty Acid Oxidation in Lymphocyte Populations of Myalgic Encephalomyelitis/Chronic Fatigue Syndrome

Jessica Maya, Sabrina M. Leddy, C. Gunnar Gottschalk, Daniel L. Peterson, and Maureen R. Hanson

## Supplementary Material

**Supplementary Table S1**. Study subject features with Bell Activity Scale score and 36-item short-form survey data by cell type with p-values.

| | | Natural Killer Cells | | | CD4+ T Cells | | | CD8+ T Cells | | |
|---|---|---|---|---|---|---|---|---|---|---|
| Collection site (n) | | ME/CFS* | Healthy controls** | P-value | ME/CFS* | Healthy controls** | P-value | ME/CFS | Healthy controls | P-value |
| Simmaron – Incline village, NV | | 15 | 10 | - | 15 | 14 | - | 26 | 17 | - |
| Ithaca College – Ithaca, NY | | 13 | 14 | - | 14 | 12 | - | 18 | 20 | - |
| Weill Cornell – NYC, NY | | 0 | 1 | - | 0 | 0 | - | 0 | 1 | - |
| John Chia Clinic – Torrance, CA | | 0 | 1 | - | 0 | 0 | - | 0 | 1 | - |
| **Race** | White | 17 | 16 | - | 17 | 15 | - | 23 | 21 | - |
| | Black or African American | 0 | 0 | - | 0 | 0 | - | 0 | 1 | - |
| | Asian | 1 | 0 | - | 1 | 1 | - | 1 | 0 | - |
| | American Indian/Alaska Native | 0 | 0 | - | 0 | 0 | - | 0 | 0 | - |
| | 2+ races | 0 | 0 | - | 0 | 0 | - | 0 | 0 | - |
| | Unknown | 10 | 10 | - | 11 | 10 | - | 20 | 17 | - |
| **Ethnicity** (Non-Hispanic/ Unknown) | | 14/14 | 14/12 | - | 16/13 | 14/12 | - | 21/23 | 20/19 | - |
| **Bell Activity Scale** | | 30.6 ± 16.2 (n=18) | 98.1± 4.0 (n=16) | 6.74E-07 | 37.0 ± 17.7 (n=18) | 96.5 ± 6.1 (n=17) | 4.42E-07 | 32.7 ± 14.1 (n=24) | 98.7 ± 3.5 (n=23) | 4.26E-09 |
| **SF-36** | n | 21 | 15 | - | 21 | 16 | | 28 | 22 | - |
| | Physical function | 33.8 ± 24.4 | 89.0 ± 24.1 | 1.10E-05 | 39.8 ± 25.3 | 83.4 ± 29.0 | 1.12E-04 | 31.6 ± 18.1 | 87.9 ± 24.1 | 1.71E-07 |
| | Role - physical | 0 | 93.3 ± 14.8 | 4.33E-07 | 6.0 ± 15.6 | 90.6 ± 20.2 | 3.90E-07 | 0 | 96.6 ± 11.7 | 1.75E-09 |
| | Body pain | 35.4 ± 22.3 | 87.6 ± 12.1 | 1.74E-06 | 42.7 ± 24.2 | 89.2 ± 14.3 | 6.59E-06 | 37.3 ± 21.5 | 91.2 ± 12.4 | 2.28E-08 |
| | General health | 20.3 ± 11.9 | 75.0 ± 14.0 | 4.71E-07 | 25.5 ± 12.7 | 70.5 ± 13.1 | 4.23E-07 | 18.1 ± 11.8 | 76.1 ± 15.5 | 2.10E-09 |
| | Vitality | 16.7 ± 20.2 | 74.0 ± 17.8 | 1.61E-06 | 17.4 ± 21.6 | 65.6 ± 16.7 | 3.16E-06 | 15.9 ± 19.6 | 69.1 ± 19.0 | 1.82E-08 |
| | Social functioning | 24.4 ± 24.5 | 91.1 ± 18.6 | 1.37E-06 | 26.8 ± 28.0 | 88.3 ± 15.3 | 1.38E-06 | 22.3 ± 23.7 | 92.3 ± 16.5 | 5.74E-09 |

**Supplementary Table S1**. cont.

| | | Natural Killer Cells | | | CD4+ T Cells | | | CD8+ T Cells | | |
|---|---|---|---|---|---|---|---|---|---|---|
| | | ME/CFS | Healthy controls | P-value | ME/CFS | Healthy controls | P-value | ME/CFS | Healthy controls | P-value |
| SF-36 | n | 21 | 15 | - | 21 | 16 | | 28 | 22 | - |
| | Role - emotional | 52.4 ± 51.2 | 94.4 ± 15.0 | 0.04 | 61.9 ± 47.5 | 89.6 ± 23.5 | 0.12 | 58.3 ± 49.4 | 85.6 ± 25.4 | 0.11 |
| | Mental health | 68.2 ± 21.7 | 85.5 ± 14.3 | 0.004 | 69.5 ± 21.1 | 77.0 ± 12.5 | 1.59 | 68.3 ± 21.1 | 76.5 ± 14.5 | 1.87 |
| | Physical component score | 22.6 ± 7.3 | 51.8 ± 7.1 | 6.05E-07 | 25.2 ± 8.6 | 51.0 ± 8.2 | 1.27E-06 | 21.7 ± 7.5 | 54.2 ± 7.1 | 2.51E-09 |
| | Mental component score | 42.5 ± 13.5 | 55.1 ± 8.7 | 5.37E-19 | 43.3 ± 11.7 | 51.5 ± 8.8 | 0.03 | 43.1 ± 12.0 | 51.1 ± 9.1 | 0.01 |

*PBMCs from the same ME/CFS subject collected before and after treatment were used for the NK and CD4+ cell flux analysis.

**PBMCs from the same control subject collected at three different times were used in the NK cell flux analysis, and two different time points were represented in the CD4+ cell flux analysis.

**Supplementary Table S2**. Antibodies and dyes used for confocal microscopy and flow cytometry.

| Dye/Target | Clone | Fluorophore | Catalog # | Company |
|---|---|---|---|---|
| Hoechst 33342 | NA | NA | H21492 | Thermo Fisher Scientific |
| eBioscience™ Fixable Viability Dye eFluor™ 506 | NA | eFluor 506 | 65-0866-14 | Thermo Fisher Scientific |
| CD3 | UCHT1 | Alexa Fluor 700 | 56-0038-42 | Thermo Fisher Scientific |
| CD4 | SK3 | Pacific Blue | 344620 | BioLegend |
| CD8 | RPA-T8 | APC Cy7 | 557760 | BD Biosciences |
| CD27 | O323 | PE-Cy7 | 25-0279-42 | Thermo Fisher Scientific |
| CD28 | CD28.2 | Super Bright 600 | 63-0289-42 | Thermo Fisher Scientific |
| CD45RA | HI100 | Super Bright 702 | 67-0458-42 | Thermo Fisher Scientific |
| CD56 | TULY56 | Super Bright 600 | 63-0566-42 | Thermo Fisher Scientific |
| CD16 | 3G8 | Super Bright 702 | 67-0166-42 | Thermo Fisher Scientific |
| BODIPY 558/568 C12 | NA | Bodipy 559/568 nm | D3835 | Thermo Fisher Scientific |
| CPT1a Rabbit mAb | D3B3 | NA | 12252S | Cell Signaling |
| Goat anti rabbit IgG (H+L) Secondary Ab | NA | Alexa Fluor 488 | A-11008 | Thermo Fisher Scientific |
| CD36 | eBioNL07 (NL07) | PerCP eFluor 710 | 46-0369-42 | Thermo Fisher Scientific |

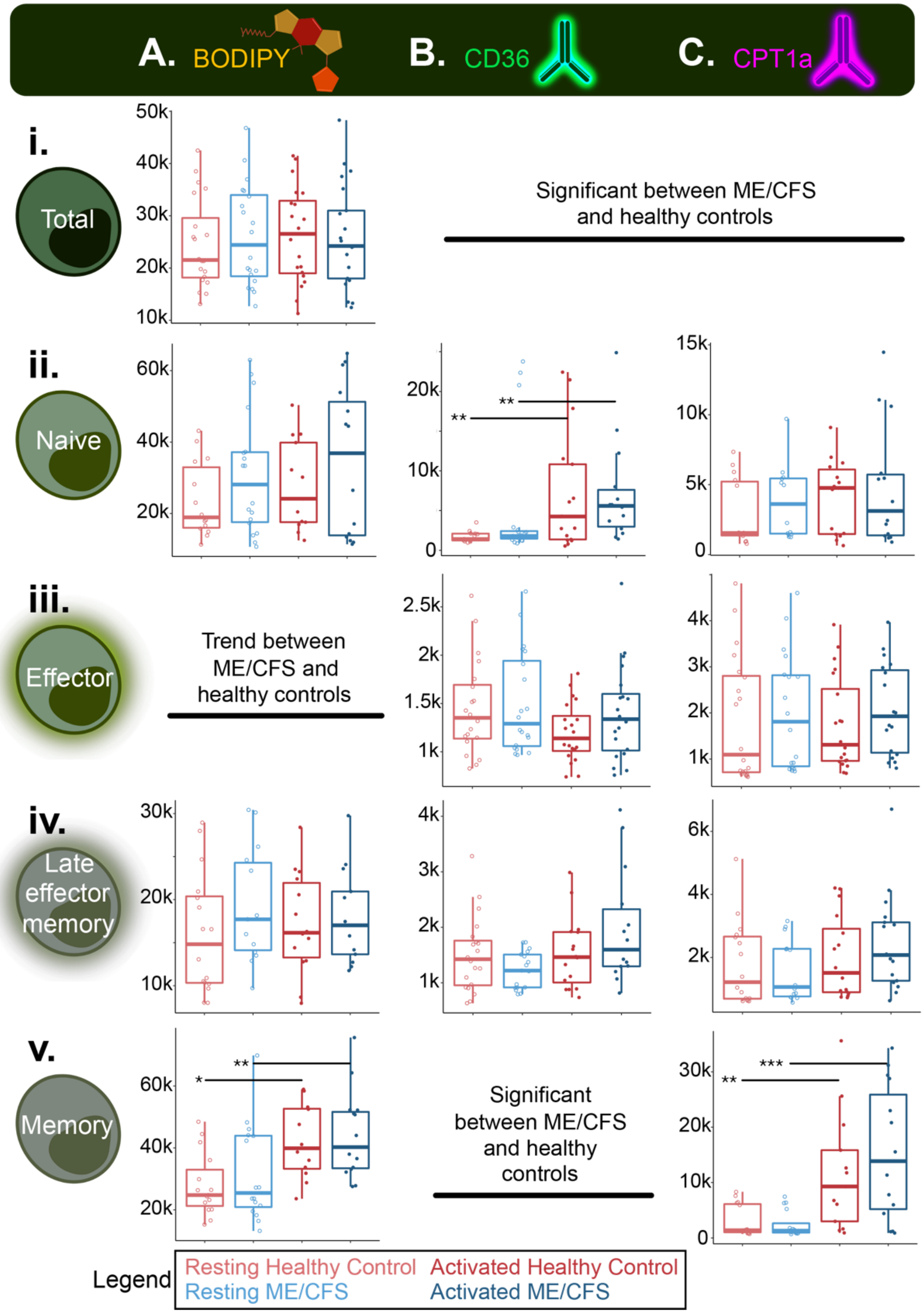


**Supplementary Figure S1.** Flow cytometry analysis of 3 fatty acid oxidation markers in all CD4+ T cell populations. MFI of (**A**) supplied fatty acid (Bodipy), (**B**) CD36, and (**C**) CPT1a in (**i**) total, (**ii**) naïve (CD27+CD28+CD45RA+), (**iii**) effector (CD27-CD28-CD45RA+/-), (**iv**) late effector memory (CD27-CD28-CD45RA+), and (**v**) memory (CD27+CD28+CD45RA-) CD8+ T cells, excluding graphs where data was significant between ME/CFS samples and healthy control samples, which can be found in Figure 5. Box plots represent the median ± 25th and 75th quartiles. Whiskers represent 1.5x the interquartile ranges. Outliers are values outside the whisker range, and *p < 0.05, **p < 0.01, and ***p < 0.001 by Kruskal-Wallis, followed by Dunn's test with FDR-based multiple testing correction.

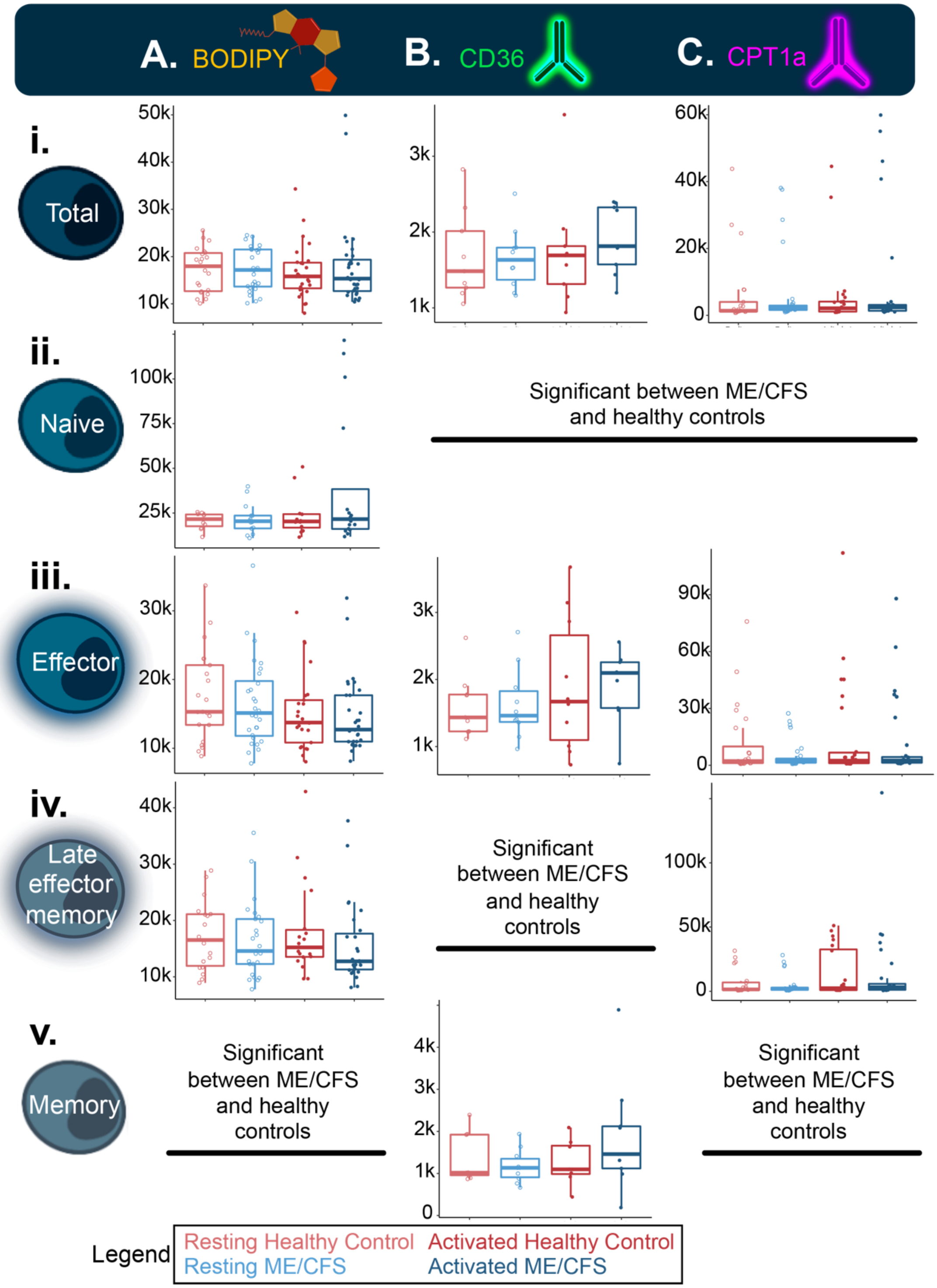


**Supplementary Figure S2.** Flow cytometry analysis of 3 fatty acid oxidation markers in all CD8+ T cell populations. MFI of (**A**) supplied fatty acid (Bodipy), (**B**) CD36, and (**C**) CPT1a in (**i**) total, (**ii**) naïve (CD27+CD28+CD45RA+), (**iii**) effector (CD27-CD28-CD45RA+/-), (**iv**) late effector memory (CD27-CD28-CD45RA+), and (**v**) memory (CD27+CD28+CD45RA-) CD8+ T cells, excluding graphs where data was significant between ME/CFS samples and healthy control samples, which can be found in Figure 6. Box plots represent the median ± 25th and 75th quartiles. Whiskers represent 1.5x the interquartile ranges. Outliers are values outside the whisker range, and *p < 0.05, **p < 0.01, and ***p < 0.001 by Kruskal-Wallis, followed by Dunn's test with FDR-based multiple testing correction.
